# Best influential spreaders identification using network global structural properties

**DOI:** 10.1038/s41598-021-81614-9

**Published:** 2021-01-26

**Authors:** Amrita Namtirtha, Animesh Dutta, Biswanath Dutta, Amritha Sundararajan, Yogesh Simmhan

**Affiliations:** 1 Department of Computational and Data Sciences, Indian Institute of Science, Bangalore, 560012 India; 2 Department of Computer Science and Engineering, National Institute of Technology Durgapur, Durgapur, West Bengal 713209 India; 3 Documentation Research and Training Centre (DRTC), Indian Statistical Institute, Bangalore, 560059 India; 4 Dept of Applied Mathematics and Computational Sciences, PSG College of Technology, Coimbatore, 641004 India

**Keywords:** Computer science, Information technology

## Abstract

Influential spreaders are the crucial nodes in a complex network that can act as a controller or a maximizer of a spreading process. For example, we can control the virus propagation in an epidemiological network by controlling the behavior of such influential nodes, and amplify the information propagation in a social network by using them as a maximizer. Many indexing methods have been proposed in the literature to identify the influential spreaders in a network. Nevertheless, we have notice that each individual network holds different connectivity structures that we classify as complete, incomplete, or in-between based on their components and density. These affect the accuracy of existing indexing methods in the identification of the best influential spreaders. Thus, no single indexing strategy is sufficient from all varieties of network connectivity structures. This article proposes a new indexing method *Network Global Structure-based Centrality* (*ngsc*) which intelligently combines existing kshell and sum of neighbors’ degree methods with knowledge of the network’s global structural properties, such as the giant component, average degree, and percolation threshold. The experimental results show that our proposed method yields a better spreading performance of the seed spreaders over a large variety of network connectivity structures, and correlates well with ranking based on an SIR model used as ground truth. It also out-performs contemporary techniques and is competitive with more sophisticated approaches that are computationally cost.

## Introduction

A spreading process is a common and natural phenomenon in various fields such as social, biological, chemical, electrical, and many others^1^. In a complex network, *influential spreaders* act as maximizers or controllers^2^ of a spreading process. Thus, an influential spreader is an essential element for a spreading process. For instance, to maximize the information spreading an influential spreader acts as a maximizer^3^, whereas, as a controller, an influential spreader can control epidemics such as COVID-19 or mitigate fake news in a social system^4–7^. Many methods have been proposed in literature to identify the influential spreaders. Pei et al.^8^ have classified those methods into *finding individual influencers* and *finding multiple influencers* categories. The *finding individual influencers* category ranks the individual nodes in a network, while the *finding multiple influencers* category finds the minimal number of nodes for maximizing the total collective influence. All the centrality approaches, such as, degree centrality^9^, kshell decomposition method^10,11^, k-truss decomposition method^12^, closeness centrality^13^ , betweenness centrality^14^, eigenvector centrality^15^, pagerank centrality^16^, expected force method^17^, hybridrank^18^, and improved gravitational method^19^ come under the first category. On the other hand, optimal percolation method^20–22^, voterank^23^, linear threshold model (LTM)^24^, and independent cascade model (ICM)^25^ are a few popular methods that belong to the second category. The first category identifies the spreading initiator and controller of a dissemination process^26–35^, and the initiator nodeset is used as an influence maximizer of a spreading process. The second category identifies the spreading initiators (i.e., minimal nodes) of a dissemination process.

We believe that the centrality approaches, which fall in the first category, are more reliable to measure the spreading capability of the nodes. Using these approaches, we can determine each node’s spreading capability, based on which we can maximize or control a spreading process from any topological position. We, therefore, study the centrality method in detail for efficient identification of influential spreaders as an influence maximizer.

Researchers have proposed various centrality measures. Most of these are the extensions of either *degree centrality* or *kshell decomposition* method^36^. Degree centrality^37,38^ is designed using the node degree. Here, the highly connected hub nodes can be identified as the best spreaders and these nodes may be located at the periphery of a network. The spreading effect of the peripheral nodes is minimal^39^. Because almost all the nodes in this region have less connectivity, the spreading cannot flow properly across the network. Further, many extensions of degree centrality have been proposed^40–43^. Among them, Kitsak et al.^39^ have shown that the most influential spreaders might not be identified from the highly connected node (decided by degree centrality) or from the most central node (decided by betweenness centrality) of a network. The authors argue that the most efficient spreaders are located at the core of a network, which is measured by the kshell decomposition method. This method follows the procedure of a global centrality approach to retrieve the core location from a network. A *global centrality approach* takes account of a node’s global position in a network relative to other nodes while calculating the rank of that node. Kshell decomposition method is a widely used method and many extensions of this method^32,43–49^ have been proposed in the literature to improve the ranking monotonicity^32^ and the spreading performance of kshell.

Pei et al.^50^ note that a node’s degree and kshell index have their own importance for measuring the influence of the nodes of a network. They have shown that the best spreaders are always located in the core of a network and determined by the kshell decomposition method, labeled as *ks*. But when the network does not have a connected structure, the best spreaders are identified using the sum of neighbors’ degree method and labeled as $k_{sum}$.

**Figure 1:**
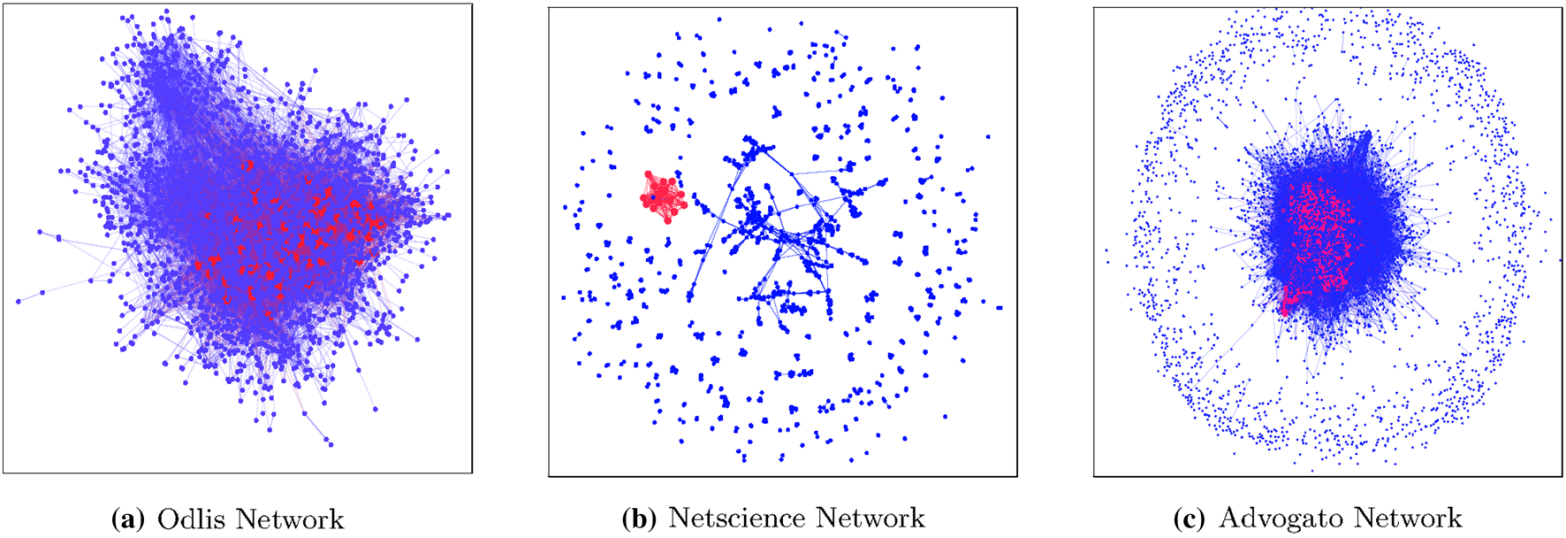
Graphical visualization of three real networks using the Gephi Tool^54^.

To examine this further, we visualize and analyze the different network connectivity structures and identify the location of the best spreaders using the kshell decomposition method. Each network’s connectivity structure is influenced by two factors: the number of connected components and the density of the network. Based on these, we can consider the network as *complete* connectivity, *incomplete* connectivity or *in-between connectivity*. Figure 1 shows the connectivity of three real networks, namely, Odlis^51^, Netscience^52^, and Advogato^53^. The *Odlis* is a library and information science network, *Netscience* is a co-authorships scientist network, and *Advogato* is a social network. It is apparent from Fig. 1a that the Odlis network has nodes form a single component with dense vertex degree, giving it a complete connectivity structure. The Netscience network (Fig. 1b) holds many disjoint sub-graphs and the nodes are not well connected by the edges, and has an incomplete connectivity structure. The connectivity of the Advogato network falls in-between in terms of connectivity. It consists of one dominant sub-graph, known as the *giant component*, where the nodes are tightly coupled by edges, but also has many small disjoint sub-graphs (Fig. 1c). In these same figures, the red colored nodes denote the best spreaders using the kshell decomposition method. We observe that for Odlis and Advogato, the best spreaders returned by kshell method are located at the dense location of those networks, whereas, for the Netscience network, kshell returns the best spreaders as being located in a separate disconnected component. Therefore, for incomplete networks, kshell performs poorly as it is a global measure. In such networks, local measures such as $k_{sum}$ will perform better.

Furthermore, Namtirtha et al.^55^ have extended the work of Pei et al.^50^ and observe that a network structure can span different connectivity ranges. Therefore, it is difficult to decide at what percentage of network connectivity—between single and disjoint components, and dense and sparse structures—that the performance of either *ks* or $k_{sum}$ will be best. They have proposed the *weighted kshell degree neighborhood method* which considers *ks*, *k*, and tunable parameters to yield a good spreading performance for the different network connectivity ranges. However, a limitation of their work is that it needs to run the simulation process multiple times to obtain the optimal value of the tunable parameter for a network. Another gap is that the authors did not study the impact of the network component structure on the spreading behavior.

Due to the different varieties of network structures as illustrated in Fig. 1, a single indexing strategy will not yield the best result in all the networks. Thus, we address the research problem: *“is it possible to design an indexing method which is based upon the network’s global connectivity structure”*. Based on our literature review, there have not been any indexing methods proposed which use the properties of the global connectivity structure of the network, which includes both the network’s components and its density, for indexing the nodes. Madotto et al.^56^ have characterized the networks based on Laplacian spectrum and the best spreading dynamics, but have not considered the global structural properties in their proposed indexing method, meta-centrality.

In this article, we propose an indexing method that will take the prior knowledge of a network’s global connectivity structure for indexing the nodes. Specifically, we find that the *network’s components* and *network’s density* are the two important characteristics of a network’s global connectivity structure, which directly influence the spreading dynamics of the network. To measure these factors, we use it global structural properties, such as the giant component, percolation threshold, and average degree. Using these global properties, we design a novel indexing method *Network Global Structure-based Centrality* (*ngsc*) to quantify the influence of the nodes. To verify the effectiveness of the proposed method in identifying the best spreaders, we examine twelve real networks and use Susceptible-Infected-Recovered (*SIR*)^7,27^ epidemic model as a benchmark for validating the influence of these nodes. The experimental results reveal that our proposed method is better able to identify the most influential spreaders compared to existing methods. Also, we observe that the our method can efficiently find the spreaders for a varieties of network structures.

The rest of the article is organized as follows. The *Methods* section describes the proposed *ngsc* method, which is the main contribution of this article; the *Results* section discusses the experimental setup and empirical results; and lastly, we offer our conclusions in the *Discussion* section.

## Methods

This section consists of three subsections. *Background Analysis* discusses the relationships between the network connectivity structure and the indexing methods. *Proposed Method* introduces the design of the proposed Network Global Structure-based Centrality (*ngsc*). *Analysis of Network Structural Properties* examines how the value of the tunable parameters are decided based on the network’s global structural properties.

### Background analysis

To solve the above-stated problem, we first study the various global connectivity structures of networks (Fig. 1) and different types of indexing strategies. Based on the study, we classify the network structure into three categories: complete connectivity, incomplete connectivity, and in-between connectivity. A network with complete connectivity is a densely connected network where all the nodes have a path to each other. An incomplete network means that it is disjoint and has a low density of connections. An in-between connectivity means the network has a combination of dense and sparse connectivity with dominant (but not just one) components.

Since in a *complete network* all the nodes are part of a single connected component, the global centrality methods such as kshell decomposition, closeness centrality, betweenness centrality, etc. perform better. The global centrality methods work well when each node can access the topological information about all the other nodes in the graph, which is possible only when there is a path from every node to every other node. Among all the global centrality methods, the kshell decomposition method (*ks*) performs better than others^39^.

However, the global centrality methods do not yield a satisfactory result in an *incomplete network*. Because most of the nodes are disconnected in this type of network, without a path to each other, it is not possible to traverse and retrieve the topological information of the other nodes from a particular source node. Due to this disjoint nature, nodes only have access to the connectivity information of their local network component. Thus, we find that the local centrality methods such as degree centrality, sum of neighbors’ degree, cluster rank, and many others work best for a sparse network. Among these, the sum of neighbors’ degree method ($k_{sum}$) is the best choice for identifying the influential spreaders for a sparse network.

But if the network holds an *in-between connectivity structure*, we use the combined weight of the kshell decomposition method and the sum of neighbors’ degree method, and we combine the two methods we using tunable parameters. Specifically, in the proposed *ngsc* method, we use the kshell decomposition method, the sum of neighbors’ degree method and two tunable parameters to identify the nodes that are the best spreaders. These are discussed next.

### Proposed method

In this paper, we work on undirected and unweighted networks. An unweighted network is represented as a *graph* $G\langle V, E \rangle$, where *V* is the set of *vertices (nodes)* and *E* is the set of *edges*. Let $v=|V|$ be the number of vertices in the graph and $e=|E|$ the number of edges. A graph can be represented by an *adjacency matrix* $A_{[V \times V]}$, where, $a_{ij}$ denotes the element of the $A_{[V \times V]}$, $a_{ij}=1$ means there is an edge between node *i* to node *j*, and $a_{ij}=0$ means that there is no edge between *i* and *j*. To design the proposed Network Global Structure-based Centrality (*ngsc*), we combine the kshell decomposition method and a sum of neighbors degree method, as described below.

#### Kshell decomposition model (*ks*)

This is a global centrality technique to retrieve the core location of a network. The nodes with the largest kshell index denote the *core* of a network. The core nodes are the most influential spreaders and the nodes with the smallest kshell index denote peripheral nodes. The procedure of assigning the kshell index to the nodes using the kshell decomposition method is as follows.

We first remove the nodes with a degree $k=1$ (i.e., nodes with just one incident edge) from the given network and assign those deleted vertices the kshell index of $ks=1$. This continues until no more nodes with degree $k=1$ are left in the network. Next, we remove nodes with a degree $k=2$ from the updated network and assign them the kshell index of $ks=2$. We repeat this procedure iteratively until no more nodes remain in the network.

#### Sum of neighbors’ degree method ($k_{sum}$)

This approach takes into account the degree of a vertex’s neighbors. It is defined for a node *m* as:

1 $$\begin{aligned} k_{sum}(m)=\sum _{n \in N(m)} k(n) \end{aligned}$$

where, *N*(*m*) is the set of neighbors of the node *m* and *k*(*n*) denotes the degree of the node *n*.

#### Network global structure-based centrality (*ngsc*)

To identify the best spreaders for diverse types of network connectivity structures, our proposed Network Global Structure-based Centrality (*ngsc*) takes account of *ks*, *k* and tunable parameters $tune_1$ and $tune_2$. These two parameters control the weight of *ks* and *k*, respectively. We find the *ngsc* for a node *i* as follows:

2 $$\begin{aligned} ngsc(i)=\sum _{j \in N(i)} \big (tune_1 \times ks(i) + tune_2 \times k(i)\big ) + \big (tune_1 \times ks(j) + tune_2 \times k(j)\big ) \end{aligned}$$

where, *N*(*i*) is the set of neighbors of node *i*; *ks*(*i*) and *ks*(*j*) are the kshell indices of nodes *i* and *j*, respectively; and *k*(*i*) and *k*(*j*) are the degrees of nodes *i* and *j* respectively. In other words, the centrality of a vertex is influenced by the kshell and degree of that vertex, and the kshell and degree of its neighboring vertices—the latter is a proxy for $k_{sum}$. The range of the tunable parameters $tune_1$ and $tune_2$ lie between [0.0–1.0]. To decide the value of the tunable parameters, we have analyzed the network global connectivity structures and their properties, which are described below.

### Analysis of network structural properties

Two factors are essential to know the network’s global structure and how a spreading process functions: *network components* and *network density*. A spreading process depends on the number and type of network components because there should be at least one path between an originator node to other nodes in a network for its influence to propagate through the entire graph. Otherwise, the influence is limited to the local component in network that the originating node is part of. On the other hand, network density is also an important factor of a spreading process. Networks with a high average vertex degree are dense while those with a low average vertex degree are sparse. If a network is connected but sparse, it has fewer edges per vertex and this limits the number of paths along which a spreading process can transmit its influence.

Based on these two factors, one can conceive of networks that have a single or dominant connected component with a high degree density, which we term a *complete connectivity* network; networks which are disjoint with a number of connected components and a sparse density, which we call an *incomplete connectivity* network; or lastly, an *in-between connectivity* where the number of components and density fall in-between these extremes of network connectivity structure.

Further, using these two factors of network components and network density, we will measure the degree of completeness of the network structure and use these to index the nodes using the proposed *ngsc* method.

#### Network components

**Figure 2:**
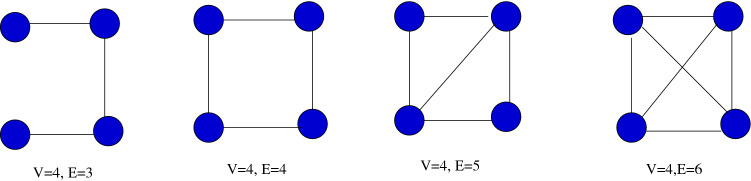
A network with a single connected component but different densities.

According to network science, a network can be of two types: *connected* and *disconnected*. A connected network means that every node has at least one path to reach all the other nodes in the network. Otherwise, the network is called a disconnected network. A connected network consists of only one connected component while a disconnected network consists of many disjoint connected components. The dominant connected component of a network is known as the *giant component*^57^ (*Gc*), and it holds a large fraction of the total number of vertices ($Gc_V\%$) and edges ($Gc_E\%$) in the network (see the example in the Supplementary document, Fig. 2). In a connected network, the sole component is the giant component and holds $100\%$ of all vertices *V* and edges *E*. Otherwise, if the giant component of the network does not have all the vertices and edges, it is a disconnected network.

In this article, we use the fraction of edges ($Gc_E\%$) present in the giant component to estimate the weight of one of the tunable parameters. The edges are the prime bridge to maximize the spreading process^58^ and without the edges, just the singleton vertices have no significance in a spreading process. To empirically estimate the $Gc_V\%$ and $Gc_E\%$ of a network, we use the Gephi visualization tool^54^.

From Note 1 of Analysis 1 in the *Supplementary document*, we find that if the $Gc\%$ (i.e., $Gc_V\%$ and/or $Gc_E\%$) tends to be high ($\approx 100\%$), then the network is inclined towards a single connected component and the kshell decomposition method works better. Whereas, if the $Gc\%$ of a network tends lower ($\ll 100\%$), then it prone to be disconnected and the $k_{sum}$ method performs well. Therefore, in the *ngsc* method, according to the value of $Gc\%$, either the weight for *ks* will be set high (we consider $90\%$ or 0.9) using $tune_1$ or the weightage given to *k* will be imposed high (we consider $90\%$ or 0.9) using $tune_2$.

Initially, it is intuitive to use $Gc_E\%$ to determine the weight of $tune_1$ or $tune_2$ as follows

3 $$\begin{aligned} tune_1&= 0.9,  if ( Gc_E\%\approx 100\%)\nonumber \\&||\nonumber \\ tune_2&= 0.9,  if ( Gc_E\% \not \approx 100\%) \end{aligned}$$

However, based on our experiments, we determine that $tune_1=0.9$ when $\{Gc_E\% \ge 97\%\}$, and $tune_2=0.9$ when $\{ Gc_E\% < 97\%\}$ perform better. We consider these limits because in a large network if most of the edges are connected but a few edges are disjoint then that network is called a disconnected network but its behavior still resembles a connected network. Further, Dorogovtsev et al.^1^ state that the second order degree distribution $\langle k^2 \rangle$, where $\langle k \rangle$ is the average vertex degree, determines the existence of a giant component in the network and this is controlled by the value of the percolation threshold ($p_c$) is:

4 $$\begin{aligned} p_c=\frac{\langle k \rangle }{\langle k^2 \rangle - \langle k \rangle } \end{aligned}$$

As a result, the percolation threshold plays an important role in determining the tunable parameters as examined later.

Further, Castellano et al.^59^ state that topological changes among the networks are depended on $\langle k^2 \rangle$ and it has a close relationship with various types of dynamics. In our experiments, we observe such a behavior among the examined networks’ $p_c$ and their spreading performance. So, we determine Eq. (3) based on our observations in Experiment 1 of the Results section reported below. Note that by using the giant component, we have decided either one of the tunable parameter’s weight, $tune_1$ or $tune_2$. The weight of the other parameter, $tune_2$ or $tune_1$, is decided by the network density.

#### Network density

Using $Gc\%$, we can identify how connected or disjoint a network is, and this gives an indication of which method, *ks* or $k_{sum}$, will have a tendency to perform better. But the performance of *ks* or $k_{sum}$ in identifying influential nodes depends on yet another factor, the density of the network. The network density is an important factor in network science and also in spreading dynamics. It is a measure of the *average degree of the nodes* in the network, i.e., the average number of edges incident on a vertex. Changing the density of a network with even a single connected component changes its structural behavior as shown in Fig. 2.

In this article, we use the network’s *average node degree* property $\langle k \rangle$ as a measure of its density. We say a network is *fully connected* when it has the maximum possible average degree, $(v-1)$, where *v* is the number of vertices. The network density can be measured by the following density function in Eq. (5):

5 $$\begin{aligned} Density=\frac{e}{e_{max}} \quad where \quad e_{max}=\frac{v\times (v-1)}{2} \end{aligned}$$

where *e* is the actual number of edges in a network and $e_{max}$ is the maximum possible edges in a network with *v* vertices. We use the average degree to measure the density of the network in our experiments since it is a measure of the average number of outbreaks that can emerge from each node of a spreading process. If the number of outbreaks is high, then the influence of a spreading process will increase. Otherwise, the influence flow will be limited. The *average degree* $\langle k \rangle$ of a network is defined as:

6 $$\begin{aligned} \begin{aligned} \langle k \rangle&= \frac{2 \times e}{v}, \quad \text {for an undirected network}\\ \langle k \rangle&= \frac{ e}{v}, \quad \text {for a directed network} \end{aligned} \end{aligned}$$

Since we operate over undirected and unweighted graphs, the first formulation is relevant.

The density of a network is an indication of the the network’s structural completeness. Different types of network may have different density level, such as high, low, or in-between. Networks with a high density or high $\langle k \rangle$ means that their nodes are well-connected; networks with a low density or low $\langle k \rangle$ means that their nodes are loosely-connected; and other networks fall in-between with a medium level of connectivity among the nodes.

The tunable parameter $tune_2$ serves as the weight of the average degree $\langle k \rangle$, both for the current node and its neighbors, which effectively incorporates the $k_{sum}$ of the current node. While the giant component was used to select the value of $tune_1$ or $tune_2$, we conduct Experiment 1 under the Results section to measure the value of the other tunable parameter, $tune_2$ or $tune_1$ as may be the case, using $\langle k \rangle$, based on its effect on the network spreading dynamics. Details of assigning an exact value to the tunable parameter are given below.

## Results

### Experiment design

In order to investigate the performance of the proposed Network Global Structure-based Centrality (*ngsc*) with the other competitive indexing methods, we have examined the SIR epidemic model as a benchmark simulator over twelve real networks—Ego-Facebook^60^, PolBlogs^61^, Advogato^53^, Epinions^62^, Brightkite^63^, Netscience^52^, CA-GrQc^64^, CA-HepTh^64^, CA-CondMat^64^, Cit-HepTh^65^, Cora^66^, and Celegans^67^. A description of these networks is provided in Table 1 and additional details are provided in the *Supplementary document*, Note 2 on Datasets.

**Table 1:** Describes the networks’ structural properties, where *V* represents the number of vertices, *E* represents the number of edges, $Gc_V\%$ and $Gc_E\%$ denote the percentage of vertices and edges in the giant component, respectively, $\langle k \rangle$ represents the average node degree, $\beta _{th}$ indicates the epidemic threshold of a network as measured by largest eigenvalue, $p_c$ is the percolation threshold, given by Eq. (4), and $tune_1$ and $tune_2$ denote the best tunable parameters selected for the proposed *ngsc* method.

Classification	Network	*V*	*E*	$\beta _{th}$	$p_c$	$Gc_V\%$	$Gc_E\%$	$\langle k \rangle$	$\langle k^2 \rangle$	$tune_1$	$tune_2$
Social	PolBlogs^61^	3982	6803	0.011	0.012	96.12	99.44	27.31	2219.35	0.9	0.4
	Ego-Facebook^60^	4039	88,234	0.036	0.009	99.9	99.9	43.69	4656.14	0.9	0.2
	Advogato^53^	6541	51,127	0.011	0.012	77.08	97.07	15.633	1255.84	0.9	0.4|0.2
	Brightkite^63^	58,228	214,078	0.0098	0.016	99.97	99.99	7.35	468.42	0.9	0.6
	Epinions^62^	75,879	508,837	0.004	0.005	99.99	100	13.412	1966.47	0.9	0.2
Collaboration	Netscience^52^	1589	2742	0.052	0.168	25.91	33.32	3.74	26.05	0.2	0.9
	CA-GrQc^64^	5242	14,496	0.011	0.063	92.29	96.44	5.53	93.25	0.2	0.9
	CA-HepTh^64^	9877	25,998	0.016	0.087	99.41	99.74	5.26	65.89	0.4	0.9
	CA-CondMat^64^	23,133	93,497	0.013	0.047	98.35	98.71	8.08	178.19	0.2	0.9
Citation	Cora^66^	23,166	91,500	0.031	0.044	65.27	77.74	7.70	182.30	0.2	0.9
	Cit-HepTh^65^	27,770	352,807	0.013	0.009	97.62	98.87	25.37	2697.53	0.9	0.2
Neural	Celegans^67^	2325	5110	0.037	0.026	99.71	99.88	8.94	358.49	0.9	0.4

To verify the spreading performance of the influential nodes identified by our *ngsc* method, we employ the *Susceptible-Infected-Recovered* (*SIR*) *epidemic model*^7,27^ as a reference model. This model is popular and also practical, especially in the context of the current COVID-19 pandemic. It computes the spreading capability of the nodes in a network. Initially, all the nodes are in a susceptible state *S*, except for one node that is in the infected state *I*. A susceptible (neighboring) node becomes infected ($S\longrightarrow I$) through direct contact with (i.e., being the neighbor of) the infected node, with an infection probability $\beta$. The value of $\beta$ is measured by the network’s epidemic threshold $\beta _{th}$. In our experiment, we set the $\beta$ value close to $\beta _{th}=\frac{1}{\sigma _1}$ where $\sigma _1$ is the largest eigenvalue of a network^12,39^. Further, an infected node can recover (*R*) with a recovery probability $\lambda$. We use $\lambda =0.8$ in our experiments^12,39^. We simulate a a spreading process for a given *infected source vertex*, which will terminate when no more nodes are in the infected state. In this termination state, the number of nodes present in the recovered state is called the *spreading capability* of the originator node which was initially infected. For each infected source vertex, we run 100 independent iterations to address the probabilistic nature of SIR and sum the counts of all nodes present in the recovered state after each iteration. This total count is the *score* used for identifying the influence of the originator node. Similarly, we calculate the spreading capability of the other nodes in the network using the SIR model, and use their scores to get a ranked list of all the nodes.

To measure the performance of the indexing methods in terms of best spreading dynamics, we correlate the ranked lists of the SIR reference model against each candidate indexing method. There are many correlation methods available, and among them Kendall’s Tau^68^ and Spearman^69^ correlation methods are commonly used. As one of our measures, we use the Kendall $\tau$ rank correlation coefficient^68,70^ which considers the full ranked list of an indexing method and of SIR. The correlation value lies between $\tau \in [-1, +1]$, where $\tau =+1$ indicates a high similarity between the two ranked lists and $\tau =-1$ denotes a high dissimilarity between the lists.

Specifically, if there are two ranked lists, *X* and *Y*, returned from the SIR model and the candidate indexing method, respectively, with $$|X|=|Y|=v'$$, where $$v'$$ is the number of nodes in the source nodeset. Let the pairs of ranks for a node *i* be given by $$(x_i, y_i)$$ and for a node *j* be given by $$(x_j, y_j)$$, where $$x_i,x_j \in X$$,  $$y_i,y_j \in Y$$ and  $$1 \le i,j \le v'$$. These pairs of ranks are called *concordant* if they satisfy:   $$(x_i > x_j$$ and $$y_i > y_j)$$ or $$(x_i < x_j$$ and $$y_i < y_j)$$. Alternatively, the pairs are called *discordant* if they satisfy:   $$(x_i > x_j$$ and $$y_i < y_j)$$ or $$(x_i < x_j$$ and $$y_i > y_j)$$. The pairs of ranks are neither concordant nor discordant if   $$x_i = x_j$$ or $$y_i = y_j$$. Given these, the Kendall $$\tau $$ rank correlation is defined as:

7 $$\begin{aligned} \tau =\frac{v_1-v_2}{0.5 \times v \cdot (v-1)} \end{aligned}$$

where *v* is the total number of nodes in the network, $v_1$ is the number of concordant pairs and $v_2$ is the number of discordant pairs, respectively.

In addition, for better comparative accuracy between the seed nodesets of the two ranked lists, we also use the *Recognition Rate* ($R_p$) metric^50,56^ which calculates the number of nodes present with the same rank in the ranked list of nodesets returned by SIR (*X*) and the candidate indexing method (*Y*). The Recognition Rate is defined as:

8 $$\begin{aligned} R_p= \frac{\sum \limits _{(x_i, y_i)~\forall x_i \in X, y_i \in Y}{ E(x_i, y_i) } }{ v'} \qquad where~~ E(x_i, y_i) = {\left\{ \begin{array}{ll} 1, &{} if~x_i = y_i\\ 0, &{} otherwise\\ \end{array}\right. } \end{aligned}$$

Here, $p = \frac{v'}{v}$ is the fraction of nodes present in the source nodeset from among the total number of nodes *v* in the network. We have conducted the experiments using these performance metrics to evaluate the proposed method.

### Experimental results

In this section, we report the results of three experiments that we conduct to verify and validate the performance of the proposed network global structure-based centrality (*ngsc*) method. *Experiment 1* evaluates the spreading performance of the *ngsc* method with respect to the SIR reference model. *Experiment 2* compares the $R_p$ and Kendall $\tau$ rank correlation for the proposed *ngsc* method against other contemporary indexing methods such as Degree centrality (*k*), Kshell decomposition (*ks*), Sum of neighbors degree ($k_{sum}$), Strength (*st*), Hybrid Rank (*hr*), EigenVector centrality (*ev*), K-truss (*kt*), Cluster Rank (*cr*), and PageRank (*pr*). Further, we also compare our performance with more sophisticated methods that identify the influential spreaders based on topological connection and spreading dynamics of a node: message-passing approach (*msg*)^34^, walks-based method (*wc*)^33^, and optimal dynamic message-passing approach (*od*)^35^. Lastly, *Experiment 3* examines the value of $R_p$ for different $\beta$ scales. The details of the experiments, their results and their analysis are provided below.

**Figure 3:**
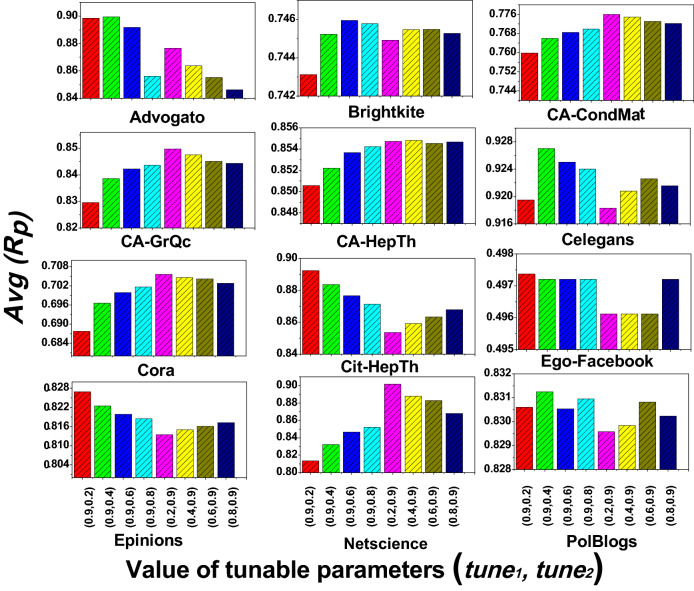
shows a bar plot of the Avg ($R_p$) for different value of tunable parameters ($tune_1$, $tune_2$).

#### Experiment 1 (determining tunable parameters)

This experiment is conducted to identify the tunable parameters that are best suited for a given network global structure, and which can result in *ngsc* performing similar to the spreading capability of the SIR model. As stated in the *Analysis of Network Structural Properties* section, the first tunable parameter is set according to the fraction of vertices and edges present in the giant component $Gc\%$ and the alternative tunable parameter is set according to the $\langle k \rangle$, i.e., if $Gc\% \approx 100\%$, then $tune_1=0.9$ and $tune_2$ is decided as per $\langle k \rangle$, whereas, if $Gc\% \not \approx 100\%$, then $tune_2=0.9$ and $tune_1$ is determined using $\langle k \rangle$.

The alternative tunable parameter, $tune_2$ or $tune_1$ as is the case, is determined from the network’s average degree $\langle k \rangle$, which depends on the network connectivity. As the connectivity of a networks can be diverse, $\langle k \rangle$ will also vary in the range of *[low, high]*, more specifically, $[0, (v-1)]$. Therefore, the weight of the tunable parameter will also span the range *[low, high]*, and in particular, [0.0, 1.0]. From this range, we select some discrete weights $\{0.2 |0.4 | 0.6 |0.8\}$ for the alternative tunable parameter and observe the spreading behavior of *ngsc* for the network:

$$\begin{aligned}&tune_2 = 0.2 | 0.4 | 0.6 | 0.8 \quad when \quad tune_1=0.9\\&tune_1= 0.2 | 0.4 | 0.6 | 0.8 \quad when \quad tune_2=0.9 \end{aligned}$$

To compare the ranking performance of *ngsc* method with the spreading dynamic SIR epidemic model we first use the *recognition rate* $$R_p$$ metric for different seed spreaders that form a nodesets. This seed nodeset is a fraction *p* of all the nodes in the network and has $$p \times v$$ vertices. We consider seed nodesets with seven different fractions, $$p \in \{0.05, 0.1, 0.15, 0.2, 0.25, 0.30,0.35 \}$$, and the $$R_p$$ is recorded for all these nodesets. Figure 3 summarizes the average of $$R_p$$, labeled as $$Avg (R_p)$$ on the Y axis, for these nodesets for different pairs of weights assigned to the tunable parameters $$(tune_1,tune_2)$$ along the X Axis, for all the evaluated networks. From the figure, we observe several clusters that arise. The best $$Avg (R_p)$$ is found with $$tune_1=0.9$$ and $$tune_2 =$$ [0.2–0.4] for the Advogato, Cit-HepTh, Ego-Facebook, Epinions, and PolBlogs networks. For the Brightkite and Celegans networks, the best $$Avg (R_p)$$ lies on $$tune_1=0.9$$ and $$tune_2 = $$[0.4–0.6]. Lastly, for the CA-CondMat, CA-GrQcm CA-HepTh, Cora, and Netscience networks, the best $$Avg (R_p)$$ if with $$tune_1 = $$ [0.2–0.4] and $$tune_2=0.9$$.

From the experimental results, we can infer some network structural behaviors. For networks with a high value of node degree $$\langle k \rangle $$ and a giant component with $$Gc\% \approx 100\%$$, the weight of $$tune_1$$ (i.e., *ks*’s weight) is high and $$tune_2$$ (i.e., *k* weight) is low when the average recognition rate $$Avg (R_p)$$ is high. For instance, the Advogato network ($$\langle k \rangle =15.633$$, $$Gc\% \approx 100\%$$), Cit-HepTh network ($$\langle k \rangle =25.37$$, $$Gc\% \approx 100\%$$), Epinions ($$\langle k \rangle =13.412$$, $$Gc\% \approx 100\%$$) and Ego-Facebook network ($$\langle k \rangle =43.69$$, $$Gc\% \approx 100\%$$) satisfy these conditions (see Table 1) and offer the best recognition rate when $$tune_1=0.9, tune_2 = $$ [0.2–0.4]. On the other hand, for the Brightkite and Celegans networks, with $$\langle k \rangle \approx $$ [7–10] and $$Gc\% \approx 100\%$$, the spreading rate of *ngsc* ranked nodes best matches the ranking by the SIR model with $$tune_1=0.9, tune_2 = $$[0.4–0.6]. Thus, we can conclude that if $$\langle k \rangle $$ is lower for a network then $$tune_2$$ should be increased for it, when $$Gc\% \approx 100\%$$.

However, from Fig. 3, we also observe a contradictory behavior for the CA-CondMat and CA-HepTh networks, where $$Gc\% \approx 100\%$$ but $$tune_1 = $$ [0.2–0.4] and $$tune_2=0.9$$ offer the best recognition rate. To find out the reason for this, we identify another network property, *percolation threshold* $$p_c$$, for the giant component. This threshold value is closely related to the existence of the giant component^1^. The $$p_c$$ value of the examined networks is given in Table 1. We can observe a relationship between $$p_c$$ and the best tunable parameters values of the examined networks. If $$p_c < 0.03$$ then $$tune_1$$ is high (*ks* weight) and $$tune_2$$ (*k* weight) is low for the best recognition rate; otherwise, for $$p_c > 0.03$$, $$tune_1$$ is low and $$tune_2$$ is high.

The reason for this behavior is that $$p_c$$ can more accurately define the presence of a giant component^1^. It is also related to network connectivity density because it is measured by the average degree and the second-order average degree. These two factors determine the network density as stated earlier in the *Network Components* section of Methods. Therefore, if a network is dense, its $$p_c$$ will be low. For example, for Advogato, Cit-HepTh, Ego-Facebook, Epinions, and PolBlogs, the $$p_c$$ values are nominal as their $$\langle k \rangle $$ and $$\langle k^2 \rangle $$ are high. In these networks with a dense connectivity structure, the spreading performance of *ks* is better than *k* and so their nodeset ranking with the best spreading is found for $$tune_1=0.9$$ and $$tune_2 = $$ [0.2–0.4]. Whereas, for all the collaboration networks including Cora, the $$p_c$$ value is high ($$p_c>0.03$$) and their density is low. So their best spreading is identified for $$tune_1 = $$ [0.2–0.4] and $$tune_2=0.9$$. Therefore, $$p_c$$ is an important property for calculating the weights of the tunable parameters. Hence, we further modify the Eq. (3) as:

9 $$\begin{aligned} \begin{aligned} tune_1&= 0.9,  if~ (Gc_E\% \approx 100\%) ~and~ (p_c<0.03)\\&||\\ tune_2&= 0.9,  if~ (Gc_E\% \not \approx 100\%) ~and~ (p_c>0.03) \end{aligned} \end{aligned}$$

On the other hand, we observe yet another pattern of behavior when the network has $$Gc\% \not \approx 100\%$$ and its $$\langle k \rangle $$ is low. In this scenario, the value of $$tune_1$$ is found to be low and $$tune_2$$ to be high when the recognition rate is high. For instance, in the CA-GrQc, Cora and Netscience networks where $$\langle k \rangle =$$ [3–6] and $$Gc\%<97\%$$ their best $$Avg (R_p)$$ lie on $$tune_1 = $$ [0.2–0.4] and $$tune_2=0.9$$. Similarly, if $$\langle k \rangle $$ is higher then $$tune_1$$ is also found to increase if $$Gc\% \not \approx 100\%$$.

**Figure 4:**
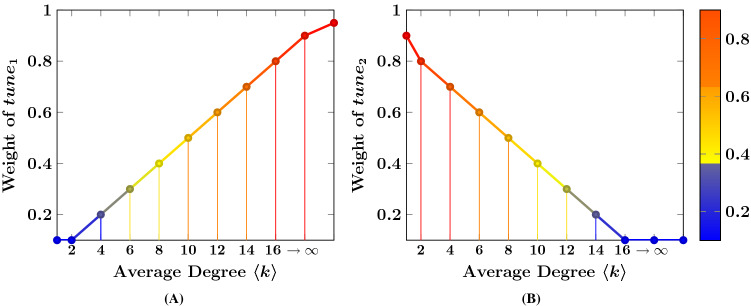
The tunable parameter’s value according to average degree (**A**) the weight of $tune_1$, (**B**) the weight of $tune_2$.

As further support, we confirm these above trends on the impact of the network’s structural behavior on the choice of the tunable parameters for *ngsc* to maximize its recognition rate by running similar experiments on a large number of networks sourced from the Sandford SNAP network repository (https://snap.stanford.edu/data/). While these results are not shown in this article, they validate our earlier observations. As a consequence of this empirical evidence, we are able to define the range of values to be assigned for the tunable parameters as a function of the average degree $\langle k \rangle$ of a network, as shown in Fig. 4, when setting the other tunable parameter as 0.9. This is the primary outcome of this experiment, which helps accurately determine the alternate tunable parameter’s value. In the figure, the X axis denotes the $\langle k \rangle$ range of $[1-\infty ]$ for an undirected network and the Y axis denotes the corresponding band for the tunable parameters $tune_1$ or $tune_2$ as is the case within the range [0.0–1.0]. The X axis ranges till $\infty$ because the network’s average degree is onbly bounded by the number of vertices and edges. Moreover, the range only starts from $\langle k \rangle =1$ since $\langle k \rangle =0$ indicates a *null graph* containing only vertices and no edges.

Figure 4A represents the weight of $tune_1$ as per $\langle k \rangle$ where $Gc\not \approx 100\%$ and $tune_2=0.9$. In the figure, the value of $tune_1$ is low when $\langle k \rangle$ is also low that means network follows incomplete structural nature in terms of the size of the giant component and density of a network. But the value of $tune_1$ simultaneously increases when the value of $\langle k \rangle$ is increased. Because if the $\langle k \rangle$ increase, that means the network is holding complete network connectivity structure in the disjoint components, therefore, in such case, the kshell method will perform better. Therefore, we increase the value of *ks* via $tune_1$.

On the other hand, Fig. 4B represents the weight of $tune_2$ as per $\langle k \rangle$ where $Gc\approx 100\%$ and $tune_1=0.9$. If a network holds $Gc\approx 100\%$ and low $\langle k \rangle$ that means network is connected but it is not structurally complete or dense. For this reason, the performance of *ks* will be decreased and $k_{sum}$ will be increased. Therefore, the weight of $tune_2$ will be assigned high. Whereas, if a network holds high $\langle k \rangle$ that means network is structurally complete and dense. Thus, the performance of $k_{sum}$ will be decreased and simultaneously $tune_2$ will also be decreased. From the above observation of $\langle k \rangle$ with the best spreading dynamic of *ngsc* method, we have estimated the weight of the second alternative tunable parameter for each mentioned networks in Table1.

**Figure 5:**
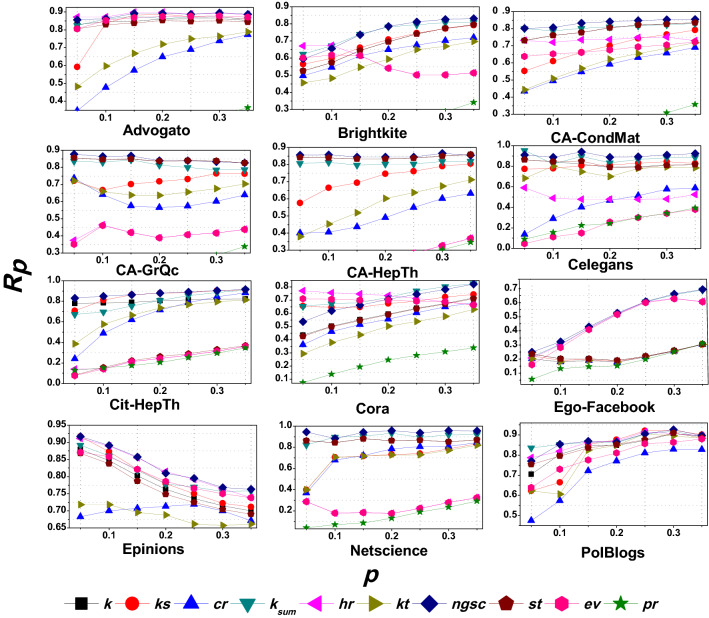
Shows $R_p$ value between the ranking list of SIR and each indexing method for all the mentioned networks with different size of source spreaders nodeset.

#### Experiment 2 (comparison with other indexing methods)

Figure 5 presents the results of a comparative study of the proposed *ngsc* method with the other contemporary indexing methods in terms of their spreading efficiency. The experiment is conducted on the twelve real networks from Table 1 and for the nine indexing methods listed above: Degree centrality (*k*), Kshell decomposition (*ks*), Cluster Rank (*cr*), Sum of neighbors’ degree ($k_{sum}$), Hybrid Rank (*hr*), K-truss (*kt*), Strength (*st*), EigenVector centrality (*ev*), and PageRank (*pr*). In the figures the X axis indicates *p* the fraction of vertices in the network that is part of the seed nodeset and the Y axis represents the recognition rate $R_p$ between the rank list of the nodes returned by SIR and the respective indexing method. The experiment is run for $p \in \{0.05, 0.1, 0.15, 0.20, 0.25,0.30,0.35\}$, and the number of nodes in each set is given by $p \times v$. Each node in the nodeset is used to start a dissemination process using the SIR model, as described earlier. The values of the tunable parameters for *ngsc* for each network are obtained from the results of Experiment 1 (Fig. 4), and are summarized in Table 1.

The experimental results reveal that our proposed *ngsc* method consistently has a higher recognition rate $R_p$ than all the other indexing methods, for all the networks evaluated at a higher fraction of nodeset *p*. In fact, it is better for *all* values of *p* for the networks CA-CondMat, CA-HepTh, CA-GrQc, Cit-HepTh, Ego-Facebook, Epinions, and Netscience, and matches the best for *most* except $1-2$ small values of *p* for Advogato, Brightkite, Celegans, and PolBlogs networks. As we evaluate a larger portion of seed nodes in the network, the local effects of specific nodes are mitigated and our proposed approach becomes comparable to the SIR model in determining the influencer rank, with $R_p \ge 0.9$ in many cases.

The *hr* method out-performs *ngsc* for some nodesets with smaller values of *p*, and is competitive with us for others. The reason for this is that *hr* is a global centrality approach that combines neighborhood coreness with eigenvector centrality. So it performs well in dense connectivity networks such as Advogato, Epinions, Ego-Facebook, and Polblogs networks, but does not perform significantly for less dense networks such as CA-CondMat, CA-HepTh, CA-GrQc, Netscience, etc. Our proposed approach is uniformly better and achieves significant spreading performance for all types of network connectivity structures.

Other centrality methods exhibit distinctive behaviors as well. The *k*, $k_{sum}$, and *st* techniques have a high-rank correlation with SIR For CA-CondMat, CA-HepTh, CA-GrQc, and Netscience because they have sparse density and are a disconnected network. Therefore, these local centrality methods perform better. On the other hand, these methods have low-rank correlation compares to others for Celegans, Cit-HepTh, Ego-Facebook, and Epinions, where these networks have a dense connectivity. The performance of *ks* is significant for networks with good network connectivity, such as Advogato, Celegans, Epinions, PolBlogs, etc. as it is a global centrality that should work better for complete network structures. Similarly, *ev*, and *hr* are global measures and these methods have mostly performed well for networks with a complete global connectivity. The *cr* and *kt* methods are both designed based on the number of triangles in the network. Therefore, we observe that these two indexing methods often have a similar trend for their rank metrics.

**Figure 6:**
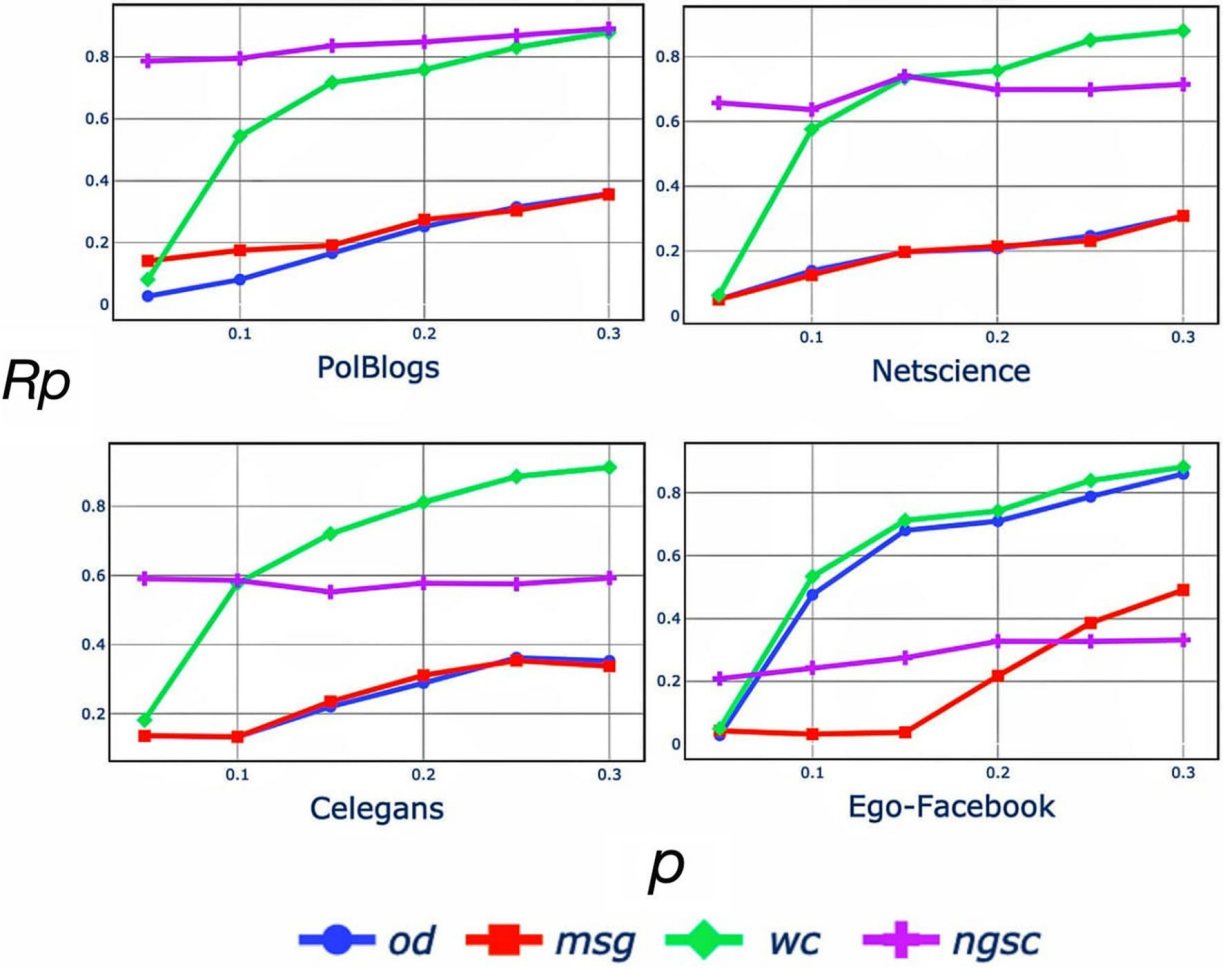
Shows $R_p$ value between the SIR and spreading dynamics related methods for Polblogs, Celegans, Netscience, and Ego-Facebook with different size of source spreaders nodeset and for 10 iterations of SIR.

Next, we perform additional experiments to compare the performance of the proposed *ngsc* method with more sophisticated methods for finding influential spreaders—message-passing approach (*msg*)^34^, walks-based method (*wc*)^33^ and optimal dynamic message-passing approach (*od*)^35^. While these methods identify the spreaders based on each node’s spreading dynamics, their time complexity is enormous and infeasible for large seed nodesets and large networks. Therefore, we perform the experiments for the small networks, PolBlogs, Netscience, Celegans, and Ego-Facebook. Figure 6 reports the recognition rate $R_p$ on the Y axis for these three new methods against *ngsc* for different values of *p* along the X axis. We observe that *ngsc* out-performs others for PolBlogs and is among the top-2 for Netscience, and Celegans—for some larger nodesets of the latter networks, *wc* performs better. For Ego-Facebook, the performance of *ngsc* is not as good. However, our proposed method is much more efficient in terms of computational time, as it only considers the network’s connectivity.

#### Experiment 3 (effect of infection probability $\beta$)

Lastly, we investigate the impact of the infection probability $\beta$ on the performance of the spreading efficiency. We measure the recognition rate $R_p$ of the proposed *ngsc* method and the other competitive indexing methods with the SIR Model for different $\beta > \beta _{th}$ values. We conduct this experiment for all the networks in Table 1, and the nine contemporary indexing methods discussed before: *k*, *ks*, $k_{sum}$, *st*, *ev*, *kt*, *hr*, *cr* and *pr*. The tunable parameters for *ngsc* are chosen based on the results of Experiment 1.

**Figure 7:**
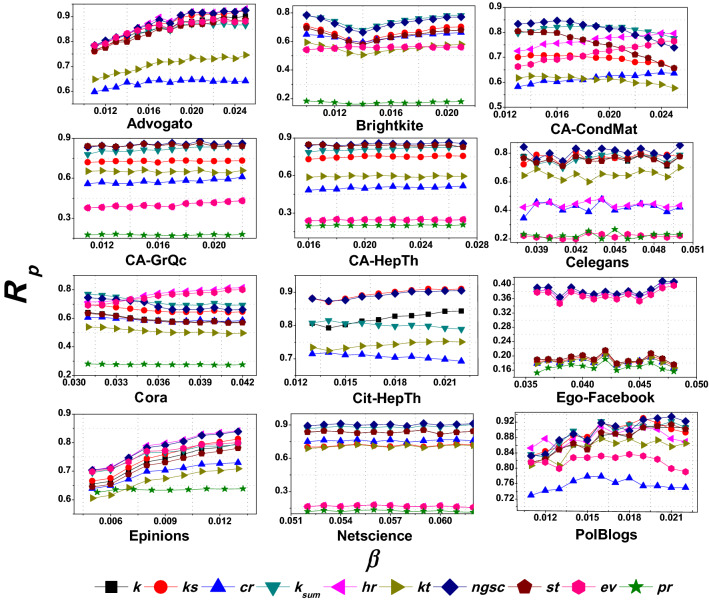
A line plot of the $R_p$ for all the mentioned networks with different infection probability $\beta$ where $\beta$ value starts from $\beta _{th}$. For this experiment, we consider $p=0.20$.

Figure 7 reports $R_p$ for different values of $\beta$. It can be observed that the recognition rate of the proposed *ngsc* method is consistently high or the best for all values of $\beta$ compared to all other methods for Advogato, Brightkite, Celegans, CA-GrQc, CA-HepTh, Cit-HepTh, Ego-Facebook, Epinions, Netscience, and PolBlogs networks. Only for two other networks, CA-CondMat and Cora, do we see that the $R_p$ of *ngsc* is higher than other methods when $\beta$ is low and close to $\beta _{th}$, but marginally deteriorates for larger values of $\beta$. In such cases, only *hr*, $k_{sum}$, and *ev* methods perform better than us. However, in a substantial majority of the networks across all values of $\beta$, the recognition rate of the *ngsc* method is among the best and exhibits strong spreading performance.

**Figure 8:**
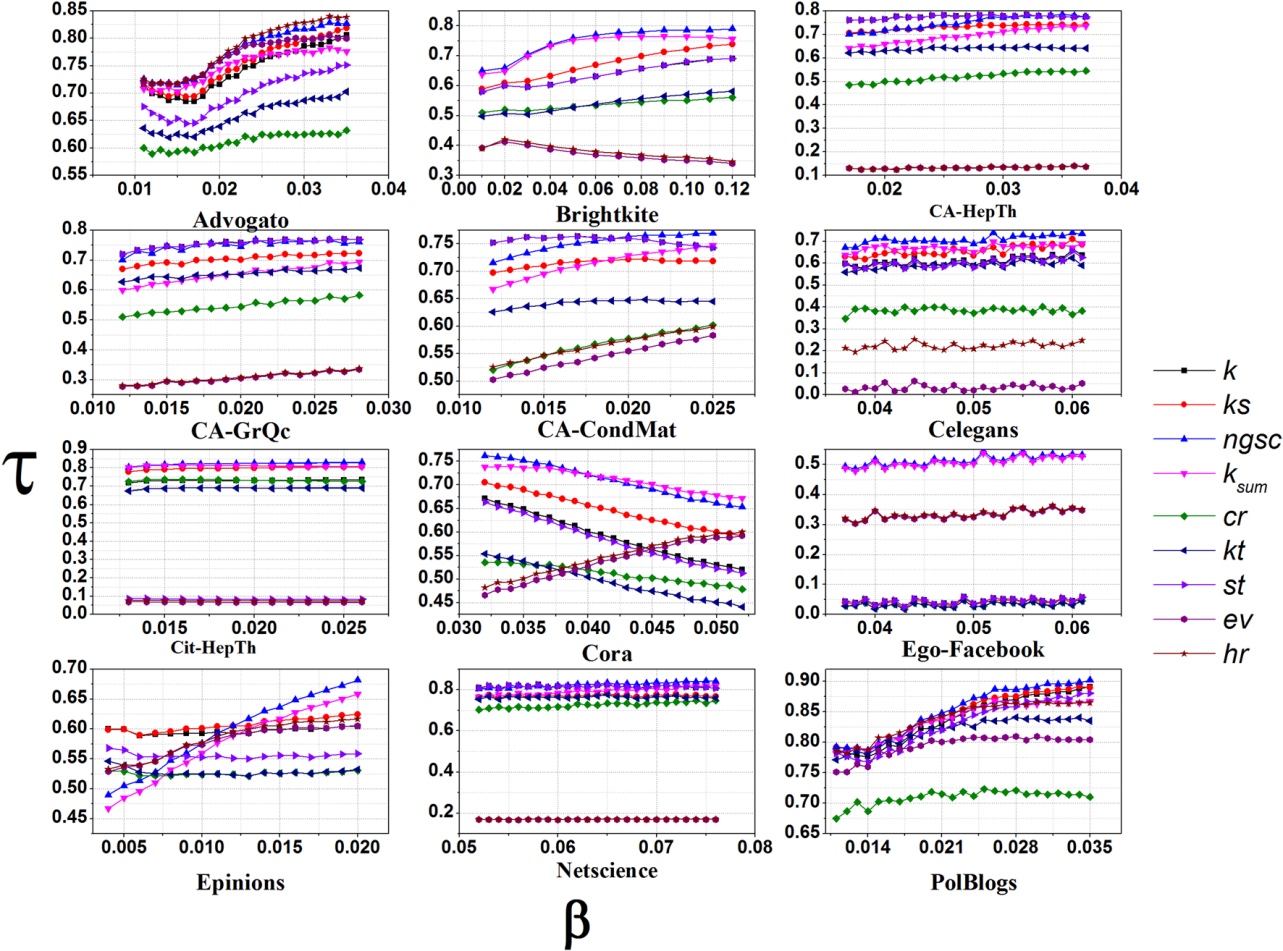
The kendall $\tau$ rank correlation between the indexing methods and SIR for different $\beta$ values.

Lastly, we compare the performance of these methods using another metric, *Kendall’s rank correlation coefficient* which determines the similarity percentage between the rank lists from the SIR model and each candidate indexing method. Figure 8 reports the result of Kendall’s $\tau$ coefficient for *ngsc* and other indexing methods. The X axis indicates the different infection probabilities $\beta$, starting from $\beta _{th}$, whereas the Y axis indicates Kendall $\tau$ correlation percentage. From the figure, we can observe that for Advogato, Brightkite, CA-GrQc, Celegans, Cit-HepTh, Cora, Ego-Facebook, Netscience, and PolBlogs networks, the proposed *ngsc* method has the best Kendall rank correlation with SIR for all values of $\beta$. For CA-CondMat, CA-HepTh, and Epinion, *ngsc* outperforms others for larger values of $\beta$ but not for smaller. This is consistent with the observations from the recognition rate metric. For some networks like for CA-GrQc and CA-HepTh, *ngsc* performs well and on par with the *st* and *k* methods. For the Advogato network, the *ngsc* and *hr* have both performed the best. It is stated in literature that indexing methods that perform best for values of $\beta$ that are slightly greater than $\beta _{th}$ are the most useful^32^. Our results indicate that *ngsc* out-performs the other methods for this range of $\beta$ and is hence significant.

## Discussion

Maximizing or controlling a spreading process in a complex network is a paramount task that benefits various fields such as social networks, epidemiology, and public transport. The major challenge in achieving this is to find the most influential spreaders within a network. In this article, first, we analyze and identify the limitations of existing indexing methods in their ability to perform well for a variety of network connectivity structures. No single strategy is resilient to such structural diversity while trying to rank the best influential spreaders. Further, we visualize and analyze the network connectivity structures of different real-world networks and identify two key characteristics that have a substantial impact on the behavior of indexing methods—network components, and network density. Based on these, we classify the networks as complete, incomplete or in-between. We note that global centrality approaches work well for complete networks while local approaches are better for incomplete ones, while no single strategy satisfies in-between networks.

We address this gap by proposing a new indexing method, *Network Global Structure-based Centrality* (*ngsc*) that attempts to leverage the best of a node’s kshell and sum of neighbors’ degree methods, which perform well for complete and incomplete networks, respectively, and tune the weightage given to each based on the network’s structure. Importantly, we are able to identify the specific metrics of a network’s giant component, its percolation threshold, and the average degree to determine the values for these tunable parameters for *ngsc* based on a detailed empirical analysis of numerous real-world graphs.

We validate our proposed indexing method based on its correlation with the influential spreaders identified by the well-respected SIR epidemic model, which provides the ground truth ranking. We use both Recognition Rate and Kendall’s Tau correlation coefficient between the ranking for the nodes returned by the SIR model and the candidate indexing methods such as *ngsc* as measures of success. Our extensive experiments compare these metrics for *nsgc* and nine other contemporary indexing methods for 12 diverse networks. The experimental results show that our proposed method is an excellent predictor in identifying the most influential spreaders from the networks. It also consistently out-performs the other compared indexing methods for various types of networks (for example, social, citation, collaboration, neural networks) and with a variety of network connectivity structures, in identifying the most influential spreaders. Additional experiments comparing *ngsc* with state-of-the-art techniques from literature that are computationally expensive show that our proposed approach is competitive in qualitative performance, while also being computationally feasible for even large scale networks.

In an epidemic model, infection probability is an important factor as the real infection rate varies from network to network, depending on the network connectivity, the interest in the topic, and many others. Hence, it is difficult to estimate the real infection rate of a network. In this study, we use the infection probability which is close to the network epidemic threshold^12,39^. Our experiments show that the *ngsc* method is robust to different values of the SIR infection probability $\beta$ and particularly yields good performance for values just above $\beta _{th}$ where SIR is most valuable.

The use of the SIR model as a ground-truth for comparison is opportune given the COVID-19 pandemic. Our proposed technique can potentially be adapted to operate over a temporal contact-graph in helping identify users who may be the most likely to spread the infection, and hence should be tested often, or be prioritized for vaccination. This is an area of active research for us. There are also other epidemic models such as SI, SIS^71^, dynamic model^72^ that exist. In the future, we aim to validate the proposed method against them.

The main limitation of the proposed method is that one of the tunable parameter’s weight is decided by the network average degree. But the procedure of assigning the weight has been experimentally determined, albeit using a large number of networks. In the future, we will continue our study to improve the design of the proposed method to better incorporate the network’s average degree value and percolation threshold of the giant component in an analytical manner with theoretical bounds. However, our proposed method is not an *optimal* solution to identify the best spreaders in a network, but an intelligent heuristic that performs well in practise. It may have a relationship with the other unknown network’s structural properties that affect the spreading dynamics. We will further explore such relationships to improve the proposed method.

## Supplementary Information

Supplementary Information 1.
