# Supplementary material for: Best influential spreaders identification using network global structural properties
Source: Sci Rep. 2021 Jan 26;11:2254. doi: 10.1038/s41598-021-81614-9 (PMC7838212; doi:10.1038/s41598-021-81614-9)
Supplement: Supplementary file 1 — Supplementary Information 1. [file 41598_2021_81614_MOESM1_ESM.pdf]

Best Influential Spreaders Identification Using Network Global Structural Properties

Supplementary Information

Amrita Namtirtha[1,*], Animesh Dutta[2], Biswanath Dutta[3], Amritha Sundararajan[4], and Yogesh Simmhan[1]

[1]Department of Computational and Data Sciences, Indian Institute of Science, Bangalore-560012, India
[2]Department of Computer Science and Engineering, National Institute of Technology Durgapur, Durgapur, West Bengal, 713209, India
[3]Documentation Research and Training Centre (DRTC), Indian Statistical Institute, Bangalore, 560059, India
[4]Dept of Applied Mathematics and Computational Sciences, PSG College of Technology, Coimbatore-641004
[*]namtirtha.asansol@gmail.com

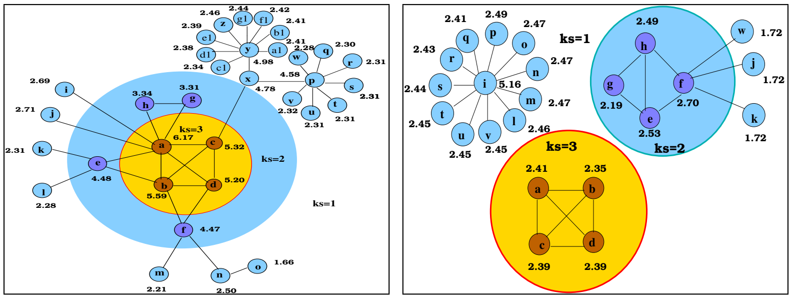

(a) Connected Network **(b)** Disconnected Network

Supplementary Figure 1. demonstrates two imaginary networks connected and disconnected respectively. The SIR epidemic model measures each node spreading capability (marked with numeric value) in the figure where infection probability β =0.30 and epidemic threshold β_{th} =0.28.

Supplementary Note 1: Analysis 1 *How the size of the giant component affects the ranking and spreading performance of ks and k_{sum} methods?*

The kshell decomposition method (*ks*) is a global centrality method. It takes account of the topological position of the others node while ranking the nodes. To do so, a network should be connected. In other words, the percentage of the giant component should be 100%, then only it can measure the topological position of other nodes. On the contrary, for a disconnected network, the measurement of topological position for all the nodes is not possible. Unlike *ks*, the k_{sum} which is a local centrality method takes account of nearest neighbors information. Therefore, it can work well for disconnected network. With the help of two schematic undirected networks Figure 1a and 1b, we show how the ranking and spreading performance of *ks* and k_{sum} depends on the size of the giant component. First, we discuss the schematic network 1a which is a connected network. Means every node has a path to reach the other nodes. This network holds only one component and it is a giant component. This giant component consists of all the vertices and edges of this network, therefore, the percentage of giant component is 100%. Next, we verify how the *ks* and k_{sum} are performing in this type of network 1a to identify the best spreaders. We apply *ks*, k_{sum} and also measure the spreading capability of each node using the SIR epidemic model (see the details in Results section). From the figure, it reveals that the best spreaders of the kshell decomposition method and SIR epidemic model are similar, i.e. $\{a,b,c,d\}$. Whereas, the best spreaders of k_{sum} method are $\{a,x,c,d\}$, where, the node x is identified from the periphery of the network and its spreading capability is less as per the SIR model. On the other hand, the second schematic network 1b is a disconnected network. It consists of three components. The largest component is the giant component which holds 52% vertices of the total number of vertex. Next, we apply *ks*, k_{sum}, and SIR model to measure the best spreaders of this network. From the figure, it is observed that k_{sum} and SIR model have identified a similar set of nodes as best spreaders i.e. $\{i,e,h,f\}$, whereas kshell has identified a different set of nodes $\{a,b,c,d\}$ as best spreaders and their spreading capabilities are less according to the SIR model. From the above analysis, we can conclude that the kshell decomposition method works well when the percentage of giant component is high, otherwise, it performs poor. Moreover, the k_{sum} performs best when the percentage of giant component is low. Therefore, we can claim that the percentage of giant component can affect the ranking of *ks* and k_{sum} methods.

Supplementary Note 2: Dataset We examine the efficiency of the proposed method on various types of real network datasets. The used network datasets have been classified into four categories: social network, collaboration network, citation network, and neural network.

Social Network: The social network represents the social relationships among the users, where the nodes represent users and the edges represent the connection between the users. We have examined with five real social networks in our experiment to evaluate the proposed method. These are (1) Ego-Facebook is a friendship network of facebook.com, (2) Polblogs network is a blogging network during US election 2004, (3) Advogato is a trusted social network for accessing the free software between the users, (4) Epinions is a social network of Epinions.com, (5) Brightkite is a location-based social network.

Collaboration Network: The collaboration network represents a scientific collaboration between the authors. If an author is a co-author of a paper with another author, then an undirected edge will be created between them. We have used four collaboration networks in the experiment. Such as (1) Netscience is a scientist co-authorship network, (2) CA-GrQc is a collaboration network of general relativity and quantum cosmology category, (3) CA-CondMat is a collaboration network of condense matter category, and (4) CA-HepTh is a collaboration network of high energy physics theory category.

Citation Network: The citation network represents the citations among the papers. If a paper i cites to paper j then a direct edge will be created from $i \rightarrow j$. We have used two citation networks in the experiment and used them as undirected networks. These are (1) Cit-HepTh covers all the citations of the papers from 1993 January to 2003 April, (2) Cora is a citation network of cora.

Neural Network-Celegans: is a neural network of frontal neurons where nodes represent neurons and edges represent synapses.

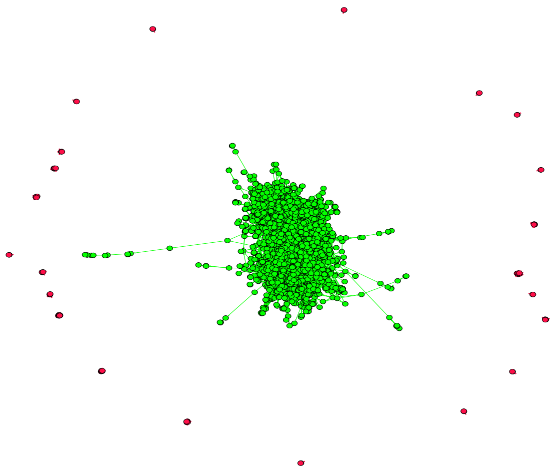

Supplementary Figure 2. shows a graphical view of Hamsterster network where the largest component is the giant component and the other components are the other disjoint components.
